# *enrichMiR* predicts functionally relevant microRNAs based on target collections

**DOI:** 10.1093/nar/gkac395

**Published:** 2022-05-24

**Authors:** Michael Soutschek, Tomás Germade, Pierre-Luc Germain, Gerhard Schratt

**Affiliations:** Lab of Systems Neuroscience, D-HEST Institute for Neuroscience, ETH Zürich, Switzerland; Neuroscience Center Zurich, ETH Zurich and University of Zurich, Switzerland; Lab of Systems Neuroscience, D-HEST Institute for Neuroscience, ETH Zürich, Switzerland; Lab of Systems Neuroscience, D-HEST Institute for Neuroscience, ETH Zürich, Switzerland; Lab of Statistical Bioinformatics, DMLS, University of Zürich, Switzerland; Swiss Institute of Bioinformatics, Switzerland; Lab of Systems Neuroscience, D-HEST Institute for Neuroscience, ETH Zürich, Switzerland; Neuroscience Center Zurich, ETH Zurich and University of Zurich, Switzerland

## Abstract

MicroRNAs (miRNAs) are small non-coding RNAs that are among the main post-transcriptional regulators of gene expression. A number of data collections and prediction tools have gathered putative or confirmed targets of these regulators. It is often useful, for discovery and validation, to harness such collections to perform target enrichment analysis in given transcriptional signatures or gene-sets in order to predict involved miRNAs. While several methods have been proposed to this end, a flexible and user-friendly interface for such analyses using various approaches and collections is lacking. *enrichMiR* (https://ethz-ins.org/enrichMiR/) addresses this gap by enabling users to perform a series of enrichment tests, based on several target collections, to rank miRNAs according to their likely involvement in the control of a given transcriptional signature or gene-set. *enrichMiR* results can furthermore be visualised through interactive and publication-ready plots. To guide the choice of the appropriate analysis method, we benchmarked various tests across a panel of experiments involving the perturbation of known miRNAs. Finally, we showcase enrichMiR functionalities in a pair of use cases.

## Graphical Abstract

**Graphical Abstract:**
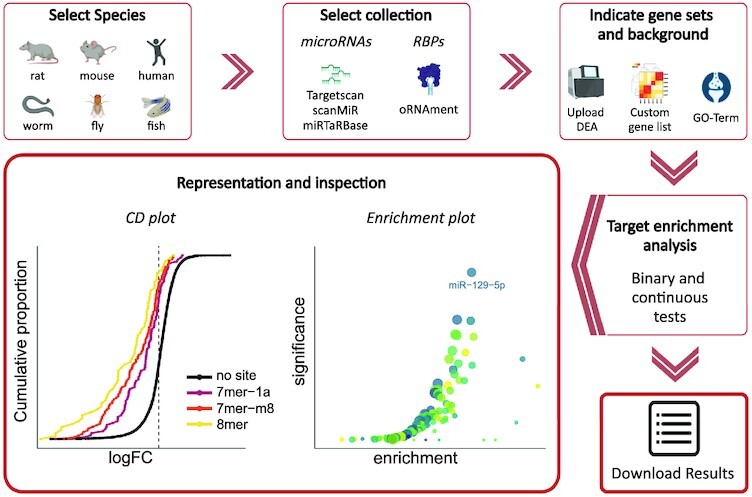
enrichMiR enables the identification of functionally relevant microRNAs from gene signatures with the help of various target collections, and offers the possibility to visualize the results in publication-ready plots.

## INTRODUCTION

Post-transcriptional gene regulation is essential to maintain cellular integrity and to precisely coordinate translational changes in response to environmental cues. microRNAs (miRNAs) and RNA-binding-proteins (RBPs) are the main post-transcriptional regulators, both impacting RNA levels and thereby controlling gene expression (1–3). Whereas miRNAs primarily exert an inhibitory role by causing mRNA silencing and degradation, RBPs regulate RNA abundance through a variety of mechanisms as they affect splicing, transport, stabilisation and localization of target RNAs (3–5).

miRNAs are a class of small non-coding RNAs that bind to complementary sequence stretches, mainly on the 3′UTR of mRNAs, as part of the RNA-induced silencing complex (RISC) (4). Notably, each of the approximately 90 broadly conserved miRNA families identified to date has an average of over 500 evolutionarily conserved canonical binding sites in human 3′UTRs (4,6), enabling systemic regulation of gene expression. There are a number of miRNA binding site collections and prediction tools that compile putative and validated miRNA-target interactions, some of which moreover ascribe scores for the strength of interaction and hence potential effectiveness of a miRNA in downregulating the potential target (7–9).

With the advent of cheap sequencing technologies and the accompanying generation of vast amounts of high-throughput data, it is becoming ever more important to develop bioinformatic tools to assist in prioritising candidates for functional follow-up experiments. Particularly, to predict the potential involvement of miRNAs, it is often useful to harness binding site collections through target enrichment analyses in given transcriptional signatures or gene-sets (10–13). Depending on the quality of the gene-set classification (e.g. the significantly changing genes in an RNA-sequencing experiment upon cellular stimulation) and the employed binding site collection, target enrichment analysis can serve as computational validation of an expected effect (14) or suggest altered activities of candidates to be explored further (15). While this approach is conceptually analogous to traditional gene-set enrichment analysis, target enrichment analysis involves additional complexities in set membership, since a given miRNA can bind a target RNA simultaneously at several sites, and different interactions can have different probabilities or intensities. There have already been several tools developed that attempt to infer the functional involvement of miRNAs in specific settings (see Supplementary Table S1). These enrichment tools can be classified into two main types: miRNA pathway association tools to test whether certain pathways are predicted to be regulated by a specific miRNA, and target enrichment tools to infer the activity of miRNAs in specified gene signatures.

Among the tools to test for miRNA associations with specific pathways (e.g. Gene Ontology (GO) or the Kyoto Encyclopaedia of Genes and Genomes), there are several examples for powerful web interfaces such as miEAA2 (16) and miRNet 2.0 (17), providing extended statistical functionalities and excellent visual representations of the results.

Likewise, several tools have been developed, based on various statistical approaches, to test for miRNA target enrichment. Early attempts to infer miRNA activity from microarray and RNA-sequencing datasets were for example conducted by Sood *et al.* (18) and van Dongen *et al.* (10), both testing for enrichments of sequence word motifs in the 3′UTRs of genes. Cheng and Li (19) as well as Arora and Simpson (20) propose other approaches, either applying rank based tests by considering gene expression levels or miRNA-target binding scores. More recently, web interfaces such as miTEA (21), mirExTra v2.0 (22) and Mienturnet (13) have been developed to make such methods more widely accessible (Table 1). Albeit offering first insights into potential post-transcriptional functionalities of miRNAs, each of these tools has serious limitations. First, most of them only enable the use of a single statistical approach (typically variations of the hypergeometric test) and some (e.g. Mienturnet) do not even offer the possibility of specifying a background or universe for the target enrichment analysis, which is notoriously problematic. Second, many of the tools are limited in their usage, especially regarding the flexibility of the inputs, such as accepting differential expression analyses in standard output formats or different gene identifiers. Moreover, only mirExTra v2.0 gives the possibility to restrict the analysis to expressed miRNAs, an essential functionality given the various cell type specific expression patterns of miRNAs (23,24). Finally, the tools mentioned do not allow specifying different plotting options of the produced results and none of them gives the option to generate cumulative distribution plots that are regarded as standard in the field to investigate regulatory effects of microRNAs.

**Table 1.** Overview of miRNA target enrichment tools and comparison of their specific web interface options

	enrichMiR	Mienturnet	mirExTra 2.0	miTEA
Statistical tests: (see Supplementary Table S1 for additional information)	A compendium of benchmarked statistical tests	Hyper-geometric test	Hyper-geometric test	Mutual minimum hyper-geometric test (mmHG)
*Web application specifications:*				
Allowed input:	Various DEA formats, custom gene lists & GO-Terms (gene symbol or Ensembl IDs)	Custom gene list (gene symbols)	Table with differentially expressed genes + background (Ensembl IDs)	Ranked gene list (gene symbols)
Supported Species:	6	6	4	5
Available binding site databases:	ScanMiR, TargetScan, miRTarBase & oRNAment	TargetScan & miRTarBase	DIANA-TarBase v7.0 & DIANA-microT-CDS	TargetScan, MicroCosm & EiMMo
Allow specific background:	Yes	No	Yes	Yes
Specify miRNA expression:	Yes	No	Yes	No
UTR length correction:	Yes	No	No	No
Plotting options:	Enrichment plots & Cumulative Distribution Plots	Network visualization	Network visualization	miRNA network visualization

Tools which used to have a web interface that is not anymore available (e.g Sylamer) are not included here. See Supplementary Table S1 for additional information on miRNA enrichment tools in general.

*enrichMiR* addresses this gap by providing a user-friendly interface to perform different statistical tests, based on various binding site collections, to rank post-transcriptional regulators (e.g. miRNAs, RBPs) according to their likely involvement in the control of a given transcriptional signature or gene-set. Inputs can be custom gene lists, Gene Ontology terms or differential expression analysis (DEA) results (all with either Ensembl ID or gene symbols). These can then be tested using different binding site databases and a collection of benchmarked statistical tests. *enrichMiR* results can subsequently be plotted as enrichment- and cumulative distribution plots or downloaded as tables including individual predicted target genes. Complementing the web interface, *enrichMiR* is in addition freely available as a documented R package to provide more experienced bioinformatic users even more flexibility in their analyses.

## MATERIALS AND METHODS

### The *enrichMiR* package and web interface

*enrichMiR* has been programmed in R and builds on a number of packages, including some specifically developed for enrichment analysis (viper and fgsea). It is available both as a standalone package and as a shiny web application. At its core is the *testEnrichment* function, offering a single flexible interface (for example adapting to various inputs and gene IDs) to various annotations, including manually curated binding site collections of, for instance, further species not provided by default in the web application. A more detailed description of the individual functionalities can be found in the package's vignette and app documentation.

### Binding site collections

*enrichMiR* provides the option to perform enrichment tests with several binding site collections. miRNA binding site predictions were obtained from TargetScan8 (http://www.targetscan.org/vert_80/) (7), scanMiR (https://bioconductor.org/packages/release/bioc/html/scanMiR.html) (9) and miRTarBase (https://mirtarbase.cuhk.edu.cn/∼miRTarBase/miRTarBase_2022/php/index.php) (8).

TargetScan annotations can additionally be restricted to conserved sites for human, mouse, rat, worm and fly. When using tests that incorporate a target score along with TargetScan predicted miRNA binding sites, the ‘cumulative weighted context++ score’ is used for human, mouse and rat predictions and the ‘total context score’ for fly, worm and fish predictions. scanMiR scores are based on the biochemical model of McGeary *et al.* (25) and were shown to predict miRNA repression efficiency more accurately than TargetScan context scores (9). However, to improve computing times, we restricted scanMiR annotations to canonical 7&8mer sites in the 3′UTR. Unlike TargetScan and miRTarBase predictions, scanMiR binding site annotations are also available at the transcript level.

As an additional feature, *enrichMiR* offers the option to perform enrichment tests for RNA-binding proteins based on the oRNAment database (http://rnabiology.ircm.qc.ca/oRNAment). However, applying *enrichMiR* in the context of RBP enrichment is rather exploratory, since the statistical tests used in the package were solely benchmarked for miRNAs.

Gene symbol to Ensembl identifier conversions were generated using the R Bioconductor biomaRt interface (26), allowing both to be used as input options for the web interface of *enrichMiR*.

### Description of the enrichment tests

*enrichMiR* implements several target enrichment tests (see Table 2 for an overview). Many of them have been previously used in this context, though often not available through a web interface (e.g. regularized regression), while some had only been applied before to other enrichment tasks (e.g. areamir, woverlap) or regression analyses (e.g. ebayes). In addition, enrichMiR implements the lmadd test, which is a variant of linear regression approaches but disentangles miRNAs with correlated regression by requiring each additional miRNA to have a significant effect conditioned on the more significant miRNAs (see the Supplementary Methods for more information). Beyond the tests themselves, we provide recommendations as to which tests should be used based on a systematic benchmark (see below).

**Table 2.** Overview of the available enrichment tests

Test	Input signal type	Annotation type	Description
overlap	binary (set)	genesets	Over-representation (ORA) of target genes among set
woverlap ^†^	binary (set)	nb sites	ORA of binding sites, correcting for UTR length
siteoverlap	binary (set)	nb sites	ORA of binding sites
areamir ^†^	continuous (DEA)	scores	Score-weighted analytic Rank Enrichment Analysis (aREA)
modsites	continuous (DEA)	nb sites	Linear regression of logFCs on nb of binding sites
modscore	continuous (DEA)	scores	Linear regression of logFCs on predicted repression scores
ebayes ^†^	continuous (DEA)	scores	Linear regression of logFCs on predicted repression scores with moderated *t*-statistics
lmadd ^†,*^	continuous (DEA)	scores	As ebayes, but followed by consecutive additive linear regression of logFCs on predicted repression scores
regmir.cc ^†^	continuous (DEA)	scores	Regularized regression to select miRNAs for linear regression testing
ks	continuous (DEA)	genesets	Kolmogorov-Smirnov (KS) test on logFCs
mw	continuous (DEA)	genesets	Mann-Whitney / Wilcoxon test on logFCs
gsea	continuous (DEA)	genesets	Gene set enrichment analysis (GSEA)

Tests denoted by an asterisk (*) are novel, while those denoted by a dagger (†) are novel in the specific context of miRNA target enrichment analysis.

The tests differ in terms of the target annotation as well as the type of input signal used for enrichment. On the signal side, tests denoted as ‘binary’ compare features (genes or transcripts) in a given set (e.g. significantly downregulated genes) to those in a background set (i.e. over-representation analysis), whereas tests denoted as ‘continuous’ instead rely on a numeric input signal, such as the magnitude or significance of changes in an input differential expression analysis (by default, the tests use the sign of the fold change multiplied by the -log10(FDR), which is well correlated to logFC for genes with low intra-group variability, and more robust than the latter for RNAseq experiments). On the annotation side, tests either use set membership (i.e. whether or not a given feature is a predicted miRNA target) or numeric values, such as the number of binding sites harboured by a given feature, or a repression score (i.e. the extent to which a given feature is predicted to be repressed by a miRNA).

### Datasets used for the benchmark as well as for *enrichMiR* use cases

Further information on the processing and use of the specific datasets can be found in the Supplementary Methods.

### Selection of the gene universe / background for *enrichMiR* analyses

Some guidance on the choice of a gene universe (background genes) for enrichMiR analyses can be found in the Supplementary Methods.

## RESULTS AND DISCUSSION

### *enrichMiR* workflow

We developed the *enrichMiR* package to allow miRNA target enrichment testing in a user-friendly and flexible way as well as to directly generate publication-ready plots of the results. In an effort to increase the accessibility of *enrichMiR* also for non-bioinformaticians, we implemented its functions in a graphical user interface web application (see the graphical abstract for a general overview of the workflow). The *enrichMiR* web app provides several pre-compiled binding site collections, currently enabling target enrichment testing for human, mouse, rat, worm, fly and fish gene signatures (see Methods and the app online help for further details). Users can supply these gene signatures in a variety of input formats: *enrichMiR* readily accepts exported ‘.csv’ files from several standard RNA-sequencing analysis algorithms (such as edgeR (27), DESeq2 (28) and others), custom compiled lists of genes of interest with the appropriate expression background as well as gene ontology annotations. If necessary, conversion between ensembl IDs and gene symbols is handled internally. The analysis can be further refined by restricting the tests to miRNAs expressed in a certain tissue of interest, which can be either indicated as a custom list, by uploading a miRNA-expression table, or by selecting one of the specific expression profiles provided for mouse and human tissues and cell types. The results of an *enrichMiR* analysis can be depicted as customizable enrichment or cumulative distribution (‘CD’) plots, including the option to change several graphical parameters. We hope this allows users to generate publication-ready plots even in the absence of any in-depth bioinformatic knowledge. In addition, *enrichMiR* provides the option to download the results of a target enrichment analysis as ‘.csv’ or Excel files, optionally comprising the annotated target genes for each regulator (see Supplementary Table S2 for an example output of an *enrichMiR* analysis).

### Visual interpretation of *enrichMiR* results

While representing the results of an *enrichMiR* analysis with an interactive ‘enrichment plot’ yields a quick overview on the ranking and potential activity of miRNAs in a certain setting (see Supplementary Figure S1A for an example enrichment plot in the context of a miRNA mimic transfection), it is often useful to inspect the behaviour of a particular miRNA more closely. This is most often done through plotting cumulative fold change distributions (CD plots). *enrichMiR* enables users who provide a DEA to generate such CD plots. By default, *enrichMiR* splits the cumulative logFC-distributions of predicted targets by the best site-type on each 3′UTR, ideally revealing a dose-response pattern along the reported different strength of various miRNA site types (29) (Supplementary Figure S1B, C). Moreover, *enrichMiR* allows splitting targets by ‘score’, distinguishing between annotated highly-effective and less-efficient binding sites (Supplementary Figure S1D). In settings without a clear signal, it can be beneficial to simply split between predicted targets and non-targets (see Supplementary Figure S1E–G for examples using different binding site collections in the context of a miRNA-mimic transfection). In general, however, we recommend the use of CD plots split by site type, as these are less prone to length biases.

### Benchmark of miRNA enrichment tests

It is well established that tremendous differences exist in the performance of various enrichment tests for gene set analysis (30,31). However, to our knowledge, the only benchmarking study in the context of miRNA target enrichment analyses is relying on microarray data, employs (meanwhile) outdated target annotations and lacks a proper assessment of false discoveries (32). Hence, to guide users in the selection of appropriate tests to perform miRNA target enrichment testing, we conducted a comprehensive benchmark on 28 RNA-sequencing datasets of miRNA-mimic transfection experiments in human cell lines as well as one tissue specific RNA-sequencing dataset of a miRNA knockout mouse model (see Methods for further details). We include in this effort several well established statistical tests and variations thereof (see Methods), as well as relevant alternatives in terms of input and annotations (see below).

**Figure 1.**
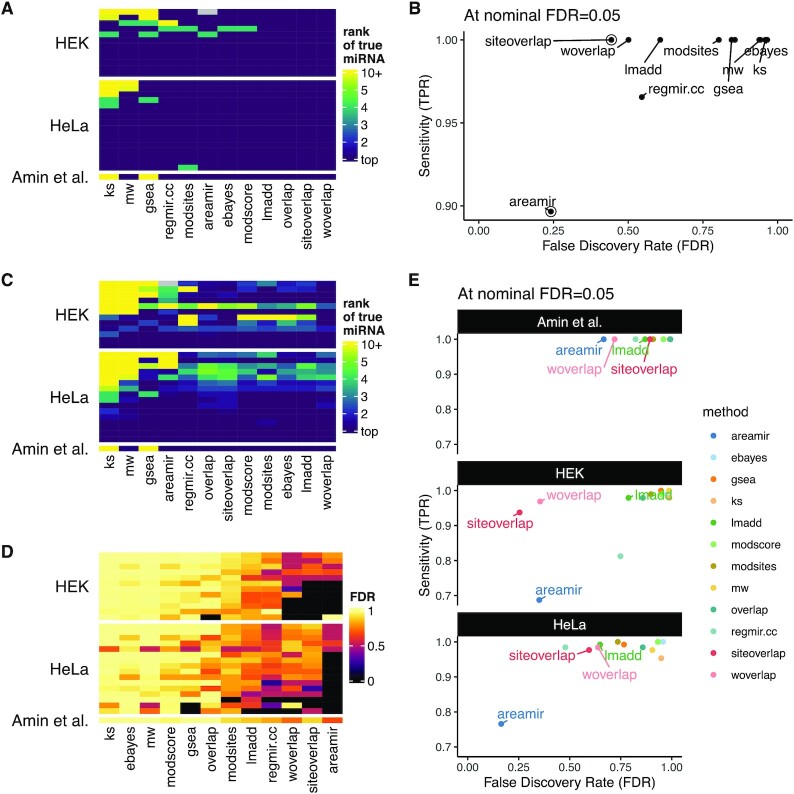
Benchmark of miRNA target enrichment tests, using the original datasets (**A**, **B**) as well as their conjunction with scrambled versions (**C–E**). (**A**) Rank of the true miRNA among the predicted candidates (the lower the better) using the various tests across the benchmark datasets. (**B**) Overall sensitivity and False Discovery Rate (FDR) at a nominal FDR threshold of 0.05, using the original datasets. (**C, D**) Rank of the true miRNA (**C**) and FDR (**D**) using the various tests across the original and scrambled datasets (averaged per dataset). (**E**) Overall sensitivity and False Discovery Rate (FDR) at a nominal FDR threshold of 0.05 across scrambled datasets.

Since the benchmark datasets were selected to ensure a signal genuinely tied to the miRNA, most of them yield a strong signal, and as a consequence nearly all methods managed to rank the true miRNA as top candidate, albeit followed by a varying number of false positives (Figure 1A, B). We therefore made the problem more difficult by generating scrambled datasets in which the expression of a fraction of target genes was randomly exchanged with that of non-target genes in each experiment (see Materials and Methods). As expected, test performances decrease steadily with the amount of scrambling (Supplementary Figure S2). Averaging over these experiments indicates differences between methods in a more comprehensive way (Figure 1C–E). While many methods still rank the true hypothesis at top for most experiments, the majority show a very high FDR. The best FDR control was achieved by the areamir method, while the siteoverlap and woverlap tests had the best sensitivity. These two methods, which take into account the fact that a given regulator can affect a target through more than one site, both outperform the classical hypergeometric test (‘overlap’) in controlling the FDR, an observation that has been similarly made by Ulitsky *et al.* (11). Of note, the GSEA method (33), which is amongst the most popular methods for traditional gene set enrichment analysis, performed rather poorly here.

It has been argued that distinguishing spliced from unspliced transcripts in RNAseq analysis could make it easier to detect miRNA-mediated post-transcriptional regulation (34). The downside of such an approach is that intron-retaining transcripts are poorly represented in typical sequenced profiles, leading to less powerful quantification and statistical analysis. We therefore tested whether using spliced (i.e. exon-specific) differential expression analysis (DEA) improved the identification of the perturbed miRNA (Supplementary Figure S3). Using exon-specific signals almost always led to a decrease in FDR, however at a cost in sensitivity which was drastic in some experiments. Of note, however, most datasets were profiled very early after transfection, diminishing the confounding effect of indirect downstream events. In other less ideal scenarios where such indirect effects are more important, the exon-specific profiles might have a larger positive impact.

We next considered whether restricting the analyses to miRNAs expressed in the given cell type improved the predictions (Supplementary Figure S4). As expected, including miRNA expression profiles again led to a decrease in FDR, at little or no cost to sensitivity. Yet, despite these clear results and the known tissue specific miRNA expression differences (23), to our knowledge, mirExTra v2.0 is the only other miRNA target enrichment tool allowing to specify expressed miRNAs.

Although it is well known that testing against a background of all annotated genes can easily create spurious enrichments, some other miRNA target enrichment web tools such as Mienturnet (13) do not enable users to specify an appropriate background. We therefore tested the extent to which using a cell-type specific background (e.g. the expressed genes) influenced the results (Supplementary Figure S5A). As expected, the use of an appropriate background had a massive positive effect on both FDR and sensitivity. Moreover, we conducted an analysis restricting the background to the highest 5000 expressed genes (Supplementary Figure S5B). While the sensitivity in this case generally decreases, the error control for some of the methods improved (e.g. for the woverlap and the areamir test).

Finally, the relative performance of individual methods might depend on the target annotation used. For example, the sets of targets per miRNA is considerably smaller in the conserved TargetScan annotation in comparison to the scanMiR binding site collection, which can naturally interfere with the performance of the tests. We therefore tested the most successful tests on both annotations (Supplementary Figure S6). Overall, enrichment analyses with the scanMiR binding site collection yielded lower performance than those based on conserved TargetScan sites. This can be partially explained by the increase in task difficulty, due to the fact that scanMiR annotations are specific for each miRNA (while TargetScan combines predicted targets of miRNA families). However, even when aggregating the predictions at the family level, scanMiR-based analyses tended to perform worse than those based on conserved TargetScan binding sites, and similarly to all TargetScan binding sites. This suggests that smaller, higher-confidence target sets are more powerful for enrichment analysis, especially for tests like siteoverlap. Of note, however, the conserved TargetScan annotation does not include all miRNAs, and for some miRNAs the target set might be too small to offer sufficient power. Further, in some cases it might be beneficial to identify an individual miRNA, instead of a miRNA family possibly comprising several members. scanMiR was shown to maximise the correlation with observed repression (9), and indeed we observed that tests relying on a regression of the input signal on predicted miRNA-mediated repression, in particular the additive linear models (lmadd), performed best in this context, achieving superior results to those obtained with the TargetScan annotation (Supplementary Figure S6). In addition, the lmadd test using scanMiR annotation could successfully identify the specific miRNA of a family in the one example of such cases among the benchmark datasets (Supplementary Figure S7).

Based on these results, we suggest testing for miRNA enrichments first using the TargetScan conserved annotation, followed by further tests using scanMiR and the lmadd statistic to obtain the most likely miRNA candidate within a given family. A summary of the benchmarking analyses is further included in the *enrichMiR* web application.

### *enrichMiR* detects a tissue specific increase in miR-7 activity upon knockout of the lncRNA Cyrano

Whereas the detection of mRNA expression changes by RNA-sequencing is by now a straightforward and affordable method, shifts in the activity of a miRNA are considerably more difficult to assess. Without a clear hypothesis one would need to perform small RNA sequencing on each tissue, leaving open the possibility that an increase in miRNA activity might not directly result in an increase in measurable miRNA levels (be it because the miRNA is relocated upon a stimulus, differently processed or loaded into the RISC, or that binding sites get more accessible). Moreover, assessing miRNA expression changes on a single-cell level is still in its infancy (35–37). Here, we demonstrate how *enrichMiR* can be used to get a first impression of potential changes in miRNA activity upon applying a certain stimulus or condition on the basis of a differential expression signature.

**Figure 2.**
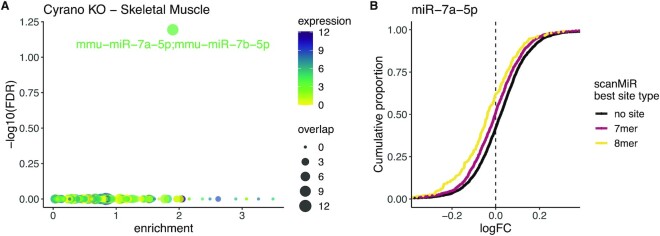
Enrichment of miR-7 binding sites in genes downregulated upon Cyrano knockout in skeletal muscle. An enrichMiR analysis was conducted on the 5’000 highest expressed genes of a DEA generated from bulk RNA-sequencing data upon knockout of the lncRNA Cyrano versus control in skeletal muscle tissue (39). (**A**) Enrichment plot showing a significant enrichment of miR-7 binding sites in downregulated genes as revealed by the siteoverlap test using the TargetScan mouse conserved annotation. Each dot depicts one miRNA family, coloured by the expression of its top expressed member in the tissue (23). The size of the dot indicates the number of predicted targets significantly downregulated in the dataset. (**B**) CD plot showing a site type dependent downregulation of miR-7a-5p targets using the scanMiR mouse annotation. Genes classified as ‘no site’ do not contain a canonical 7mer or 8mer site.

Some miRNA binding sites lead to a degradation of the bound miRNAs (a process called target RNA-directed miRNA degradation, TDMD) instead of causing post-transcriptional silencing of the target (38). One of the most prominent TDMD examples is the miR-7 binding site on the lncRNA Cyrano (39–41). Kleaveland *et al.* (39) have shown that knockout of this lncRNA in mice yields a significant increase of miR-7 levels in several tissues, followed by a downregulation of miR-7 targets in some of these tissues. By applying *enrichMiR* on each DEA of all the sequenced tissues, we predict a strong increase in miR-7 activity in cortex, hippocampus, skeletal muscle as well as moderately enhanced miR-7 activity in the striatum/thalamus (Figure 2, Supplementary Figure S8). Notably, albeit Kleaveland *et al.* (39) showed a substantial upregulation of miR-7 levels in the cerebellum, *enrichMiR* suggests, in concordance with the original study, that the effect on miR-7 targets in these areas upon Cyrano knockout are negligible. Moreover, a surprising result warranting further investigation is the potential increase in activity of the lowly expressed miR-325 in the spleen (Supplementary Figure S8).

### *enrichMiR* suggests an increase in miR-129 activity in neurons upon PTX stimulation

**Figure 3.**
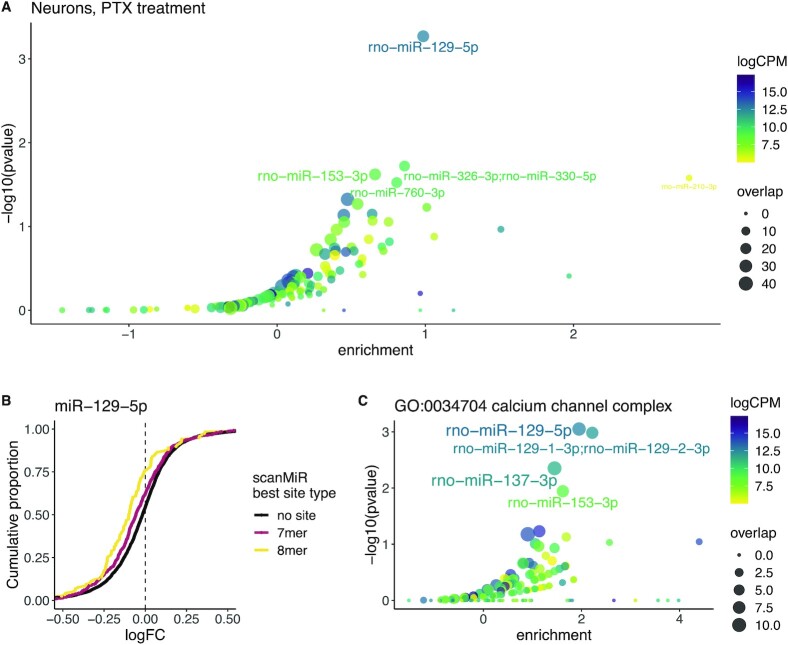
enrichMiR analysis suggests a role of miR-129-5p upon PTX treatment in neurons. An enrichMiR analysis was performed on the 5’000 highest expressed genes of a DEA generated from RNA-sequencing data of hippocampal neurons treated with PTX versus control (44). (**A**) Enrichment plot depicting the ranking of miRNAs by enrichment and p-value as assessed with the siteoverlap test on the TargetScan rat conserved annotation. Each dot depicts one miRNA family, coloured by the expression of its top expressed member in hippocampal neurons (44). The size of the dot indicates the number of predicted significantly downregulated target genes of each miRNA. (**B**) CD plot indicating a downregulation of miR-129–5p targets using the ScanMiR rat annotation (split by best site type, ‘no site’ indicating genes without a canonical 7mer or 8mer binding site). (**C**) An enrichMiR analysis of genes belonging to the GO-Term ‘Calcium channel complex’ against the same background of expressed genes as used before (44) employing the siteoverlap test and the Targetscan rat conserved annotation reveals a significant enrichment of miR-129 targets in this gene set.

It has been shown that miRNAs play a significant role in orchestrating changes in neuronal activity in response to extracellular cues (42,43). We hence wondered whether *enrichMiR* is also able to detect functional miRNA enrichment signals under such more physiological conditions, using neuron stimulation with the GABA-A receptor blocker picrotoxin (PTX) as a paradigm. Rajman and colleagues have shown that PTX stimulation for 48h leads to an increase in miR-129-5p levels, and that this miRNA is required for the PTX-mediated changes in neuronal activity mediated by synaptic downscaling (44). While an *enrichMiR* analysis on the transcriptional changes observed upon PTX stimulation in hippocampal neurons with the siteoverlap test did not yield a miRNA passing the FDR-threshold of 0.05, miR-129-5p was ranked as the top candidate (Figure 3A). This shows that, even when the signal is insufficient to provide proper statistical power, *enrichMiR* can serve as a powerful discovery tool by ranking potential candidates for further investigation. Supporting this notion, CD plots of miR-129-5p further corroborate its predicted involvement in post-transcriptional regulation of the neuronal response upon PTX-treatment (Figure 3B, Supplementary Figure S9).

Finally, we wondered whether the increase of miR-129-5p activity upon PTX-stimulation could be linked to a particular cellular component. Calcium-signalling is essential for synaptic downscaling (45), and it has been shown that miR-129-5p regulates the calcium pump Atp2b4 (44). By conducting an *enrichMiR* analysis on the GO-Term ‘Calcium channel complex’ we obtained further support for an involvement of miR-129-5p in regulating calcium signalling upon PTX stimulation in neurons (Figure 3C).

## CONCLUSION

*enrichMiR* provides a user-friendly workflow to perform miRNA target enrichment analyses in a very flexible manner, implementing various tests based on different binding site collections. Its web application enables users not especially trained in bioinformatics to conduct such analyses with a set of benchmarked statistical tests and interpret their results with the help of customizable, publication-ready plots. In addition, the downloadable plot data and R package allows more skilled users to seamlessly integrate analyses in their workflows and adapt the functions to other purposes or annotations.

Of the subset of other similar tools that have the same specific aim, i.e. miRNA target enrichment analysis, only some have a web interface enabling their use by a wide audience (Table 1). Those that do (e.g. Mienturnet, miTEA and mirExTra) have a number of major shortcomings. In particular they are mostly based on feature-level hypergeometric approaches that generally yield a high number of false positives (see Figure 1) and are as such very suboptimal for this specific task. Moreover, most other tools are very restricted in the input formats they accept, and in some cases they do not even allow the specification of a background/universe, which is critical to such approaches. Furthermore, none of the other tools enable the visualization of cumulative foldchange distribution plots, which is the gold standard to plot genuine miRNA effects. The web interface of *enrichMiR* not only addresses these gaps, but contains a number of additional features, such as preset miRNA expression collections and support for transcript-level inputs (which to our knowledge only *enrichMiR* allows so far). Finally, enrichMiR is the only tool offering access to the scanMiR binding site database, enabling to disentangle miRNAs with identical seeds by using the newly-developed lmadd test (Supplementary Figure S7).

To guide users, we provide a benchmark of the implemented tests. When the annotation consists of relatively few high-confidence targets, we find that the siteoverlap and woverlap tests perform best for binary inputs, and that the areamir test performs best for continuous input. When using broader annotations, such as those generated with scanMiR, we instead recommend the lmadd test. Of note, most tests are incorrectly calibrated, yielding a higher FDR than the one reported. This means that enrichment results should be interpreted as supportive evidence or to generate hypotheses for further studies rather than as conclusive evidence. The potential for *enrichMiR* to detect functional post-transcriptional regulators in various settings depends of course largely on the availability and accuracy of pre-compiled binding site collections. We expect those databases to get more accurate and comprehensive in the future. Due to its flexible nature, *enrichMiR* can also easily include such novel binding site collections for target enrichment analyses. We for instance included the additional option to perform RNA-binding protein (RBP) target enrichment based on the oRNAment database (46) (see Supplementary Figure S10 for an example RBP target enrichment analysis). Finally, we expect that with the generation of more detailed transcript level quantifications upon cellular stimulation, miRNA target enrichment analyses based on transcript level target annotations will become increasingly important. We hope that the *enrichMiR* suite will assist researchers, especially those with a wet-lab background, in choosing candidates for functional studies following initial screening experiments.

## DATA AVAILABILITY

The *enrichMiR* R package is available at https://github.com/ETHZ-INS/enrichMiR and the shiny web application at https://ethz-ins.org/enrichMiR/ (without login requirements). Scripts and functions used to generate the results and plots of this paper are available at: https://github.com/ETHZ-INS/enrichMiR_paper.

## Supplementary Material

gkac395_Supplemental_Files
