# Supplementary material for: enrichMiR predicts functionally relevant microRNAs based on target collections
Source: Nucleic Acids Res. 2022 May 24;50(W1):W280–9. doi: 10.1093/nar/gkac395 (PMC9252831; doi:10.1093/nar/gkac395)
Supplement: gkac395_Supplemental_Files [file gkac395_supplemental_files.zip › EnrichMiR Supplementary Material combined.pdf]

enrichMiR predicts functionally relevant microRNAs based on target collections

Michael Soutschek[1,2], Tomás Germade[1], Pierre-Luc Germain[1,3,4,*], Gerhard Schratt[1,2,*]

SUPPLEMENTARY METHODS

Further information on the enrichment tests

enrichMiR includes the option to perform the following tests, used as well for the miRNA target enrichment benchmark:

- **overlap** (binary signal, set membership):
  This test is based on Fisher's exact test, using the number of features (i.e. transcripts/genes) among predicted targets vs those in the background (and therefore ignoring any site-based information). To improve performance, *enrichMiR* uses a vectorial implementation of the test.
- **siteoverlap** (binary signal, set membership):
  The siteoverlap test is based on Fisher's exact test, but uses the number of sites on predicted targets and in the background instead of counting each feature as one. While in theory this violates the assumption of independence of the counts (since all the binding sites of a given transcript are either in or out of the set), leading to slightly anti-conservative p-values, in practice this test is excellent at identifying the most enriched miRNA.
- **woverlap** (binary signal, set membership):
  This test is like the above siteoverlap test, but corrects for UTR length (using the total number of binding sites in the collection as a proxy) through the Wallenius method, as implemented in the goseq package (1).
- **areamir** (continuous signal, score or set membership):
  The areamir test is based on the analytic Rank-based Enrichment Analysis (aREA) test implemented in the 'msviper' function of the *viper* package (2). The test is akin to an analytical version of GSEA (see below), but it can additionally use degree or likelihood of set membership. If available in the annotation, areamir will therefore use a (trimmed) version of repression scores as set membership likelihoods.
- **Mann-Whitney (MW)** (continuous signal, set membership):
  This is the Mann-Whitney (also known as Wilcoxon) non-parametric test comparing the signal (e.g. log-foldchanges) of targets and non-targets.

- **Kolmogorov-Smirnov (KS)** (continuous signal, set membership):
  This is the Kolmogorov-Smirnov test comparing the signal distribution (e.g. log-foldchanges) of targets vs non-targets.
- **modscore** (continuous signal, repression score):
  This is a linear regression testing the relationship between the input signal (e.g. log-foldchanges) and the corresponding repression score predicted for a given miRNA.
- **modsites** (continuous signal, number of sites):
  This is a linear regression testing the relationship between the input signal and the number of predicted binding sites for a given miRNA, correcting for UTR length.
- **ebayes** (continuous signal, repression score):
  This is equivalent to modscore, but testing the relationship between the input signal (e.g. log-foldchanges) and the corresponding repression score predicted for a given miRNA by using limma's empirical Bayes framework (3) and moderated *t*-tests, yielding improved FDR. This test should always be preferred to modscore.
- **lmadd** (continuous signal, repression score):
  This test is akin to the ebayes test, with two important modifications. First, the fact that the repression of different miRNAs is correlated (e.g. due to an UTR length effect) tends to create a spill-over enrichment. To adjust for this, the lmadd regression includes as a covariate the median repression score across all miRNAs (in sparse annotations, the mean is instead used). In addition, the lmadd further tests whether each miRNA significantly adds to the explanation provided by more highly ranked miRNAs. This yields highly specific predictions, especially adequate for the scanMiR annotation.
- **GSEA** (continuous signal, set membership)
  This test uses the multi-level fast GeneSet Enrichment Analysis (GSEA) implemented in the fgsea package (4), which is highly successful for Gene Ontology enrichment analysis. In the context of our benchmark, however, it performed poorly.
- **regmir**
  The regmir test uses constrained lasso-regularised regression. The test will use binary or continuous inputs (using then either linear or binomial regression), as well as binary set membership or predicted repression score, depending on the availability of the input. The continuous version, working akin to 'modscore' but simultaneously on all miRNAs and with extra regularization, worked best in our hands. For the selected coefficients, *p*-values are then computed by fitting a single linear model.

Processing of the benchmarking datasets

The benchmark datasets include 28 miRNA transfection experiments from McGeary et al. (5), of which 16 were in HeLa cells and 12 in HEK293 cells, as well as a miRNA knockout dataset in mouse neuronal samples from Amin et al. (6). Reads from human samples were mapped using STAR 2.7.3a (7) on the GRCh38.p13 genome using the Ensembl 98 annotation, while reads from mouse samples were mapped using STAR 2.6.1b on the GRCm38.p5 genome using the Ensembl 91 annotation. Only

genes with at least 10 read counts in at least 2 samples were considered for the further analysis. Standard quantification was performed using featureCounts 1.6.4 (8) with the *--largestOverlap --primary* options. Differential expression analysis (DEA) was performed using edgeR with the glmLRT test (9). For the McGeary et al. datasets, which were generated in batches, the batch was included as a covariate in the DEA. Since these datasets did not include controls, fake control samples were generated by using the median of normalized read counts from each batch.

To quantify spliced (i.e. exonic) and unspliced transcripts, we ran featureCounts with three sets of parameters: i) using exons as features (standard), ii) using exons with *--nonSplitOnly*, and iii) using transcripts as features with *--nonSplitOnly* (all three additionally shared the "*-O --largestOverlap --nonOverlap 3 --fracOverlap 0.9 --primary*" parameters). The first was used as a quantification of processed transcripts; for unprocessed transcripts, we subtracted from the third the counts of the second (which are compatible with processed transcripts as well). Post-transcriptional (i.e. exon-specific) DEA was then performed using a *~isExonic*treatment* model (additionally including the batch for the McGeary samples) and testing for the significance of the *isExonic:treatment* coefficient.

Finally, for each miRNA perturbation experiment, additional 'scrambled' experiments were created by permuting the expression values of a fraction of target genes (20%, 35% or 50%) with those of non-target genes. Each permutation was duplicated and the same across methods. Unless specified otherwise, benchmarks were run using the TargetScan conserved binding sites of the respective species.

Additional datasets

The RNA-sequencing results of cyrano-knockout vs wildtype mice, as obtained by Kleaveland et al. (2018) (10), were downloaded from the Gene Expression Omnibus server (GEO) (series GSE112415).

Transcriptomic profiling reads of rat hippocampal neurons upon PTX-stimulation were obtained from Rajman et al. (11) and mapped to the rat genome (rnor_6) using the Ensembl 99 annotation with STAR 2.6.1c. featureCounts was again used for standard count quantification and a differential expression analysis performed with edgeR. The 5’000 highest expressed genes were considered for the *enrichMiR* analysis. SmallRNA sequencing data on rat hippocampal neurons as generated and analyzed by Rajman et al. (11) was used to assess the expression of miRNAs in this setting. We considered only miRNAs expressed higher than 5 logCPM.

Mouse tissue and cell type specific miRNA expression profiles were downloaded from GSE119661 (12) and GSE30286 (13). Human miRNA expression profiles were imported from the microRNAome Bioconductor package (14).

Choice of background

The background or universe defines the null hypothesis against which the enrichment in the set of genes of interest is measured and tested. As such, it applies only to tests that are based on over-representation (e.g. siteoverlap), and not to tests that are based on a continuous signal (e.g.

areamir). In the context of over-representation analysis, the background is critical to meaningful results. An inappropriate background, in particular a background that is too broad (e.g. all genes), will often lead to spurious results.

The choice of the appropriate background depends on the circumstances, but a key consideration is what genes could have made it into your selection. For example, if your selection are genes that are differentially-expressed in a given setting, then only genes that could have been differentially-expressed should be included in the background. This means that genes which are too lowly-expressed for any differential pattern to be detected ought to be removed from the background.

In general, we recommend for initial discovery enrichment analyses, a rather lenient background selection to ensure sufficient sensitivity. When visualizing and further investigating the results though with CD-Plots, we recommend to consider only the highly expressed genes since the accuracy of the fold-change estimates increases with the genes' read counts. Depending on the cell type, intensity of the expected effect and sequencing quality, taking approximately the top 5'000 genes might be a starting point for such analyses.

Figures

The main figures were compiled using mainly R packages ggplot2 (15) and cowplot (16). The graphical abstract was created using Biorender.com and Affinity Designer.

SUPPLEMENTARY REFERENCES

1. Young MD, Wakefield MJ, Smyth GK, Oshlack A. Gene ontology analysis for RNA-seq: accounting for selection bias. Genome Biol. 2010 Feb 4;11(2):R14.
2. Alvarez MJ, Shen Y, Giorgi FM, Lachmann A, Ding BB, Ye BH, et al. Functional characterization of somatic mutations in cancer using network-based inference of protein activity. Nat Genet. 2016 Aug;48(8):838–47.
3. Ritchie ME, Phipson B, Wu D, Hu Y, Law CW, Shi W, et al. limma powers differential expression analyses for RNA-sequencing and microarray studies. Nucleic Acids Res. 2015 Apr 20;43(7):e47.
4. Korotkevich G, Sukhov V, Budin N, Shpak B, Artyomov MN, Sergushichev A. Fast gene set enrichment analysis [Internet]. bioRxiv; 2021 Feb [cited 2022 Feb 17] p. 060012. Available from: https://www.biorxiv.org/content/10.1101/060012v3
5. McGeary SE, Lin KS, Shi CY, Pham TM, Bisaria N, Kelley GM, et al. The biochemical basis of microRNA targeting efficacy. Science [Internet]. 2019 Dec 20 [cited 2021 May 17];366(6472). Available from: https://science.sciencemag.org/content/366/6472/eaav1741
6. Amin ND, Bai G, Klug JR, Bonanomi D, Pankratz MT, Gifford WD, et al. Loss of motoneuron-specific microRNA-218 causes systemic neuromuscular failure. Science. 2015;350(6267):1525–9.
7. Dobin A, Davis CA, Schlesinger F, Drenkow J, Zaleski C, Jha S, et al. STAR: Ultrafast universal RNA-seq aligner. Bioinformatics. 2013;29(1):15–21.
8. Liao Y, Smyth GK, Shi W. featureCounts: an efficient general purpose program for assigning sequence reads to genomic features. Bioinformatics. 2014 Apr 1;30(7):923–30.
9. Robinson MD, McCarthy DJ, Smyth GK. edgeR: a Bioconductor package for differential

expression analysis of digital gene expression data. Bioinformatics. 2010 Jan 1;26(1):139–40.

10. Kleaveland B, CY S, Stefano J, DP B. A Network of Noncoding Regulatory RNAs Acts in the Mammalian Brain. LID - S0092-8674(18)30634-2 [pii] LID - 10.1016/j.cell.2018.05.022 [doi]. 2018;(1097-4172 (Electronic)).
11. Rajman M, Metge F, Fiore R, Khudayberdiev S, Aksoy-Aksel A, Bicker S, et al. A microRNA-129-5p/Rbfox crosstalk coordinates homeostatic downscaling of excitatory synapses. EMBO J. 2017 Jun 14;36(12):1770–87.
12. Kern F, Amand J, Senatorov I, Isakova A, Backes C, Meese E, et al. miRSwitch: detecting microRNA arm shift and switch events. Nucleic Acids Res. 2020 Jul 2;48(W1):W268–74.
13. He M, Liu Y, Wang X, Zhang MQ, Hannon GJ, Huang ZJ. Cell-Type-Based Analysis of MicroRNA Profiles in the Mouse Brain. Neuron. 2012;73(1):35–48.
14. McCall MN, Kim MS, Adil M, Patil AH, Lu Y, Mitchell CJ, et al. Toward the human cellular microRNAome. Genome Res. 2017;27(10):1769–81.
15. Wickham H, Chang W, Henry L, Pedersen TL, Takahashi K, Wilke C, et al. ggplot2: Create Elegant Data Visualisations Using the Grammar of Graphics [Internet]. 2021 [cited 2022 Feb 21]. Available from: https://CRAN.R-project.org/package=ggplot2
16. Wilke CO. cowplot: Streamlined Plot Theme and Plot Annotations for “ggplot2” [Internet]. 2020 [cited 2022 Feb 21]. Available from: https://CRAN.R-project.org/package=cowplot
17. Lackinger M, Sungur AÖ, Daswani R, Soutschek M, Bicker S, Stemmler L, et al. A placental mammal-specific microRNA cluster acts as a natural brake for sociability in mice. EMBO Rep. 2019 Feb 1;20(2):e46429.
18. Scheckel C, Drapeau E, Frias MA, Park CY, Fak J, Zucker-Scharff I, et al. Regulatory consequences of neuronal ELAV-like protein binding to coding and non-coding RNAs in human brain. Black DL, editor. eLife. 2016 Feb 19;5:e10421.
19. Ince-Dunn G, Okano HJ, Jensen KB, Park W-Y, Zhong R, Ule J, et al. Neuronal Elav-like (Hu) Proteins Regulate RNA Splicing and Abundance to Control Glutamate Levels and Neuronal Excitability. Neuron. 2012 Sep 20;75(6):1067–80.

Supplementary Figure S1

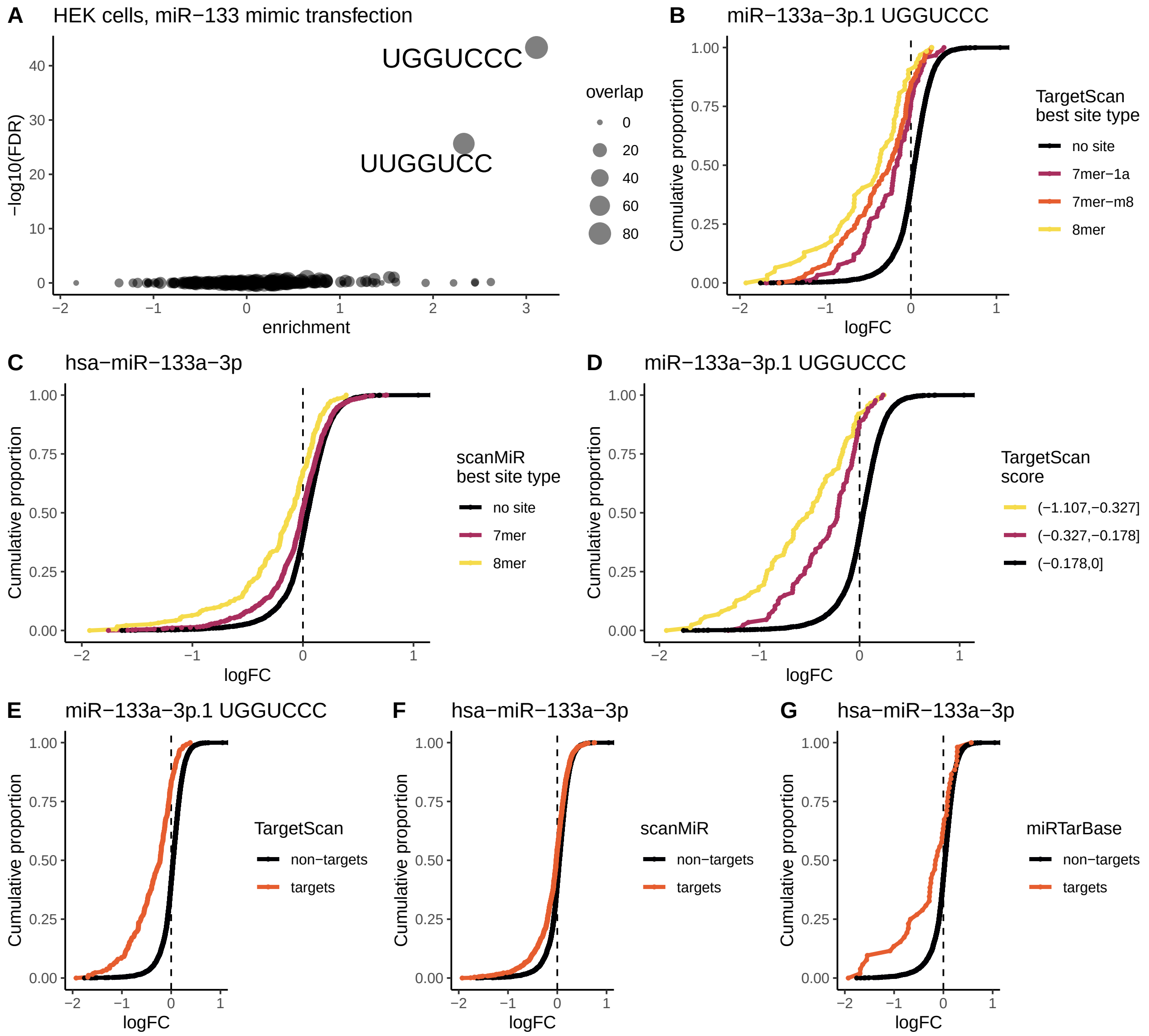

Supplementary Figure S1

Example *enrichMiR* analysis on a miR-133 overexpression dataset. A: Enrichment plot showing the results of an *enrichMiR* analysis on a DEA generated from RNA-sequencing data upon miR-133 overexpression in HEK cells using the siteoverlap test and the TargetScan human conserved annotation. Each dot depicts one miRNA family with the top two ranked candidates being paralogues of the miR-133 family ("UGGUCC" = miR-133a3p.1, "UUGGUCC" = hsa-miR-133a-3p.2/hsa-miR-133b). **B-G:** CD plots of the same *enrichMiR* analysis employing the following parameters: **(B)** TargetScan human conserved annotation & split by best site type, **(C)** scanMiR human & split by best site type, **(D)** TargetScan human conserved & split by score as well as plots **(E-G)** splitting in targets and non-targets with all three provided miRNA target collections. Genes classified as "no sites" or "non-targets" are generally not classified as miRNA-target in the respective annotation, hence those without a conserved binding site in the 3'UTR (TargetScan conserved annotation), without a canonical 7mer or 8mer site in the 3'UTR (scanMiR) or without a experimentally validated miRNA binding site (miRTarBase).

Supplementary Figure S2

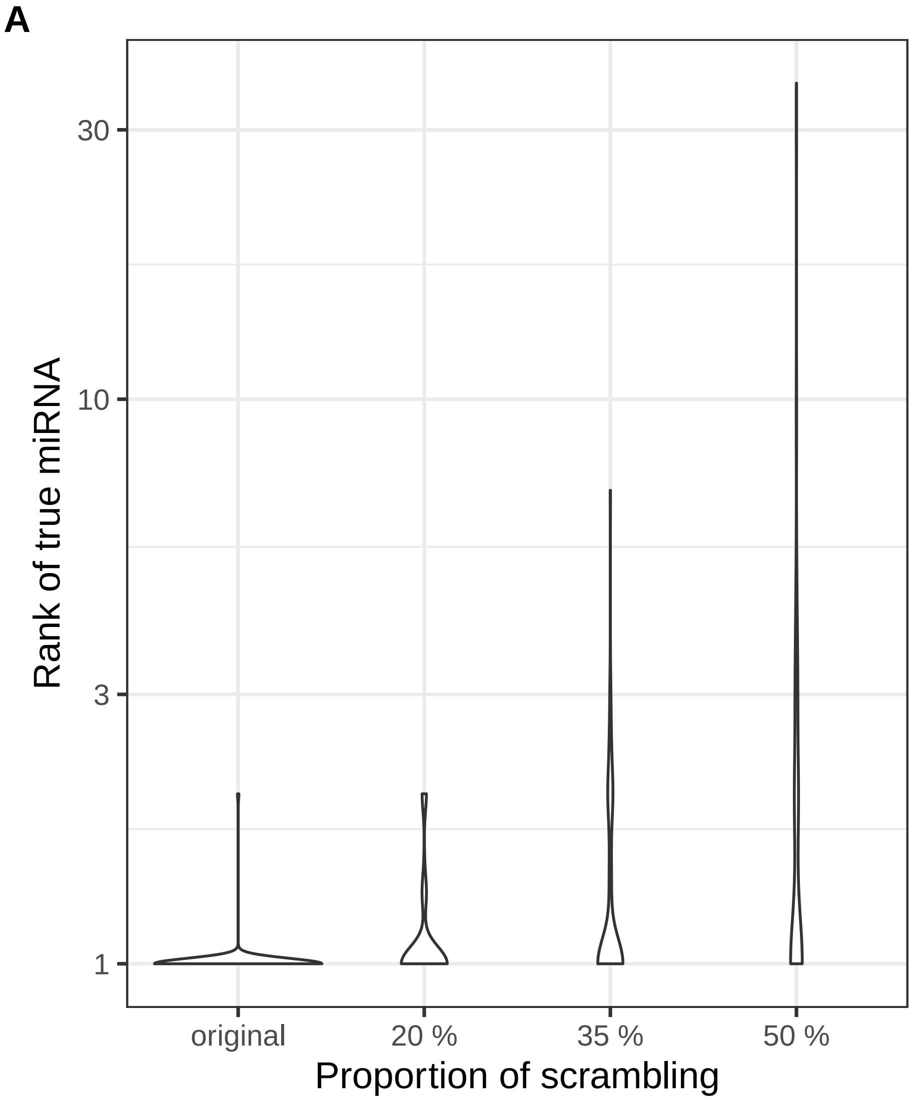

Supplementary Figure S2

Benchmark of the target enrichment methods with scrambled datasets. A: Effect of the partial scrambling on the detection accuracy.

Supplementary Figure S3

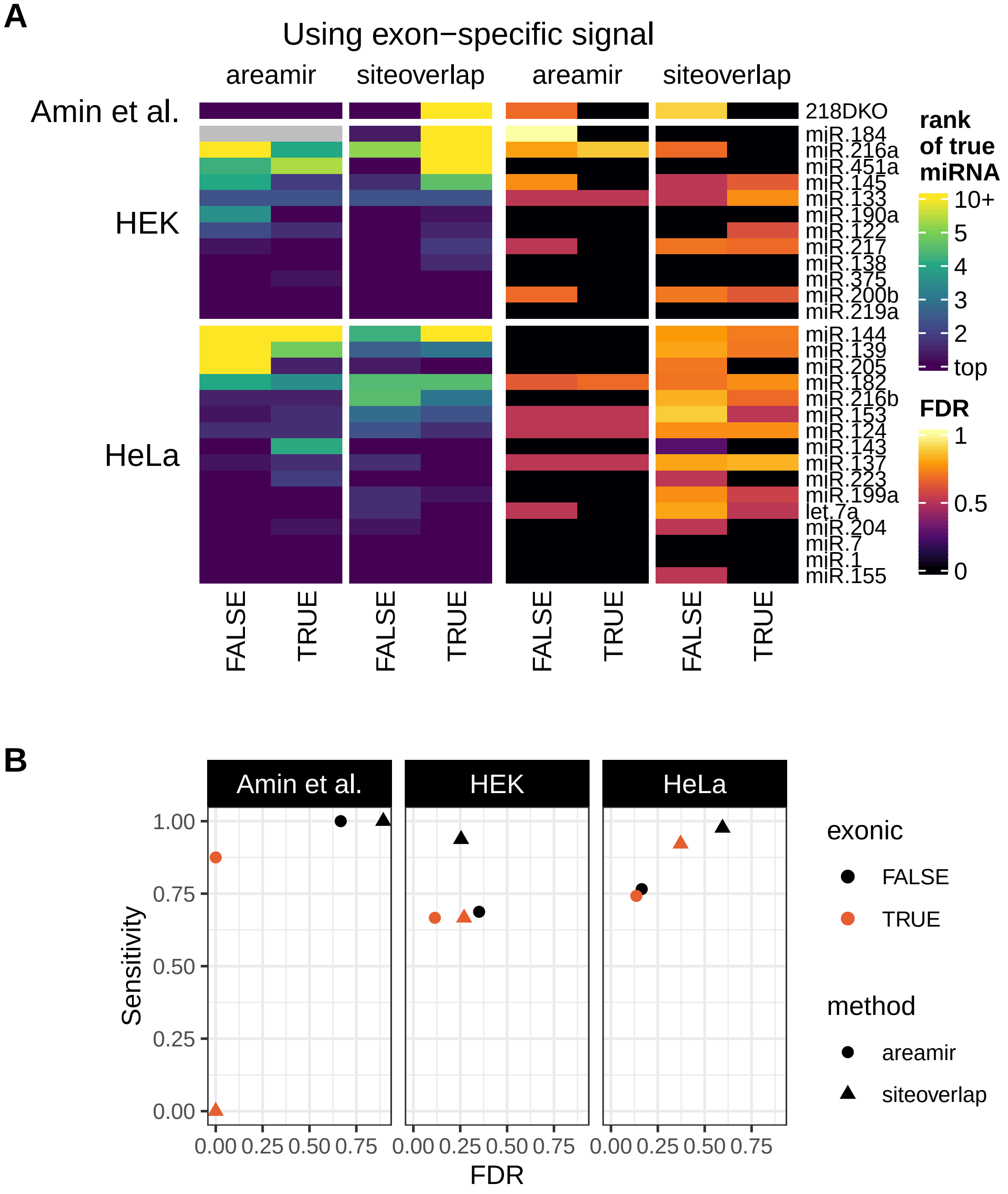

Supplementary Figure S3

The use of exon-specific signal improves FDR, but at a cost in sensitivity. A: Rank of the true miRNA (left) and FDR (right) using full or exon-specific signal, with the two best-performing tests. **B:** Summary of the specificity/sensitivity tradeoff of using exon-specific signal.

Supplementary Figure S4

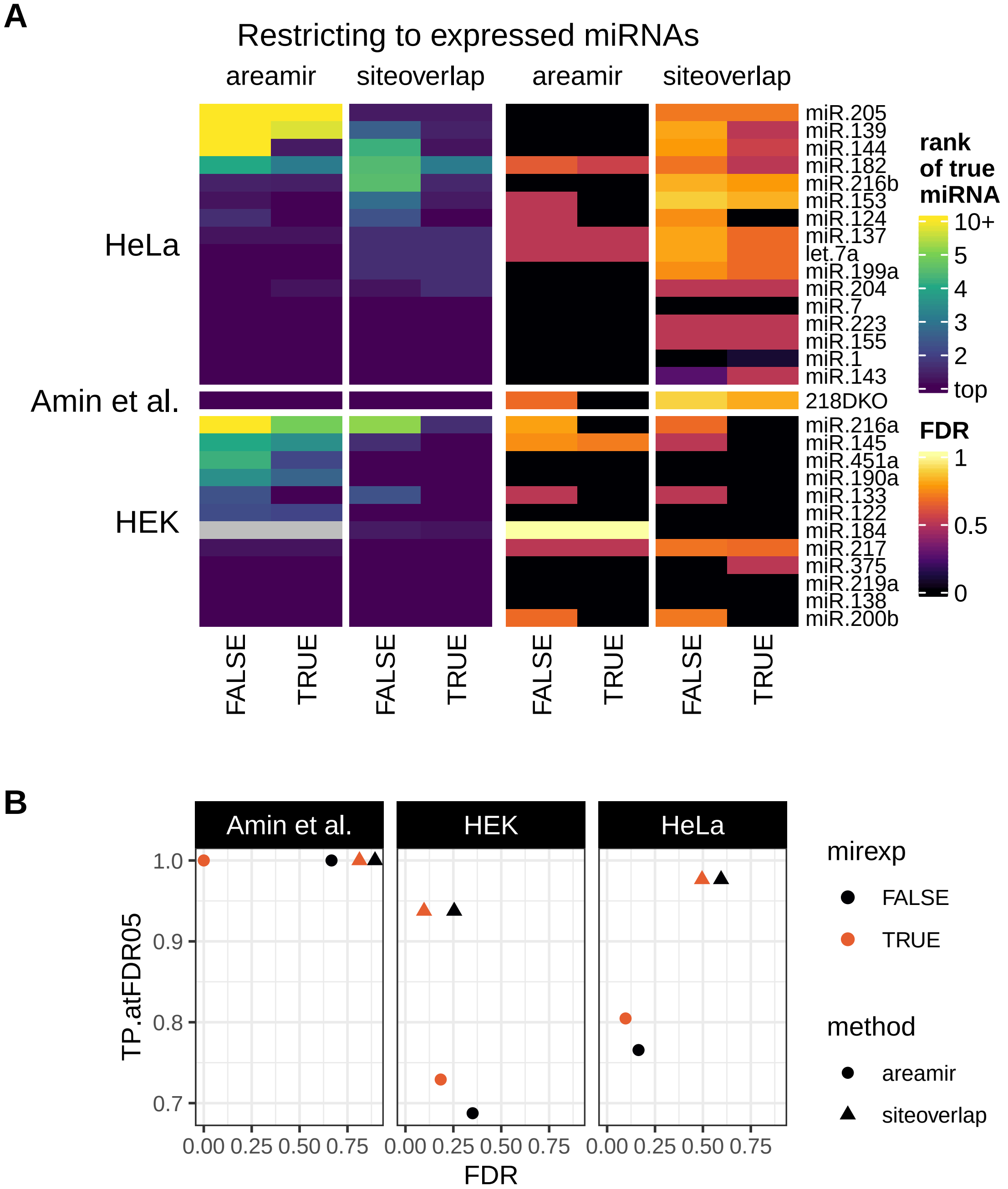

Supplementary Figure S4

Restricting hypotheses to expressed miRNAs systematically improves performance. **A:** Rank of the true miRNA (left) and FDR (right) using all or only expressed miRNAs, with the two best-performing tests. **B:** Summary of the specificity/sensitivity of using all or only expressed miRNAs.

Supplementary Figure S5

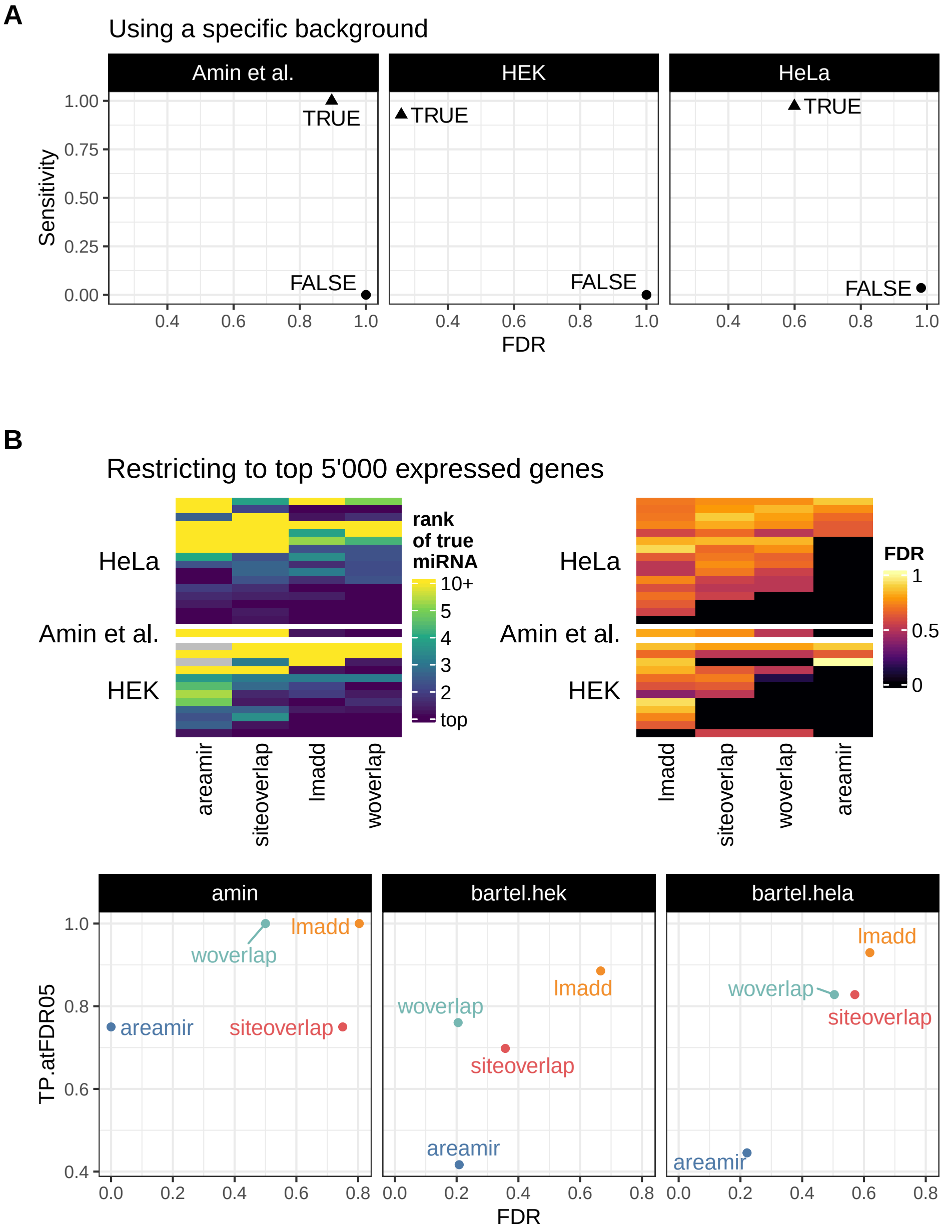

Supplementary Figure S5

Benchmark performance of the statistical tests without appropriate background specification as well as with using only the top 5'000 expressed genes. A: Impact of using a background (TRUE), i.e. the set of expressed genes, or all genes (FALSE) on the accuracy and FDR of the prediction when using set-based analysis (based on the siteoverlap test). **B:** Using the top performing tests on only the 5'000 highest expressed genes in the benchmarking datasets. As expected, due to a lower number of potentially annotated targets the sensitivity of most tests decreases. However, the relative performance of some of the tests changes (with e.g. the woverlap test being particularly more powerful).

Supplementary Figure S6

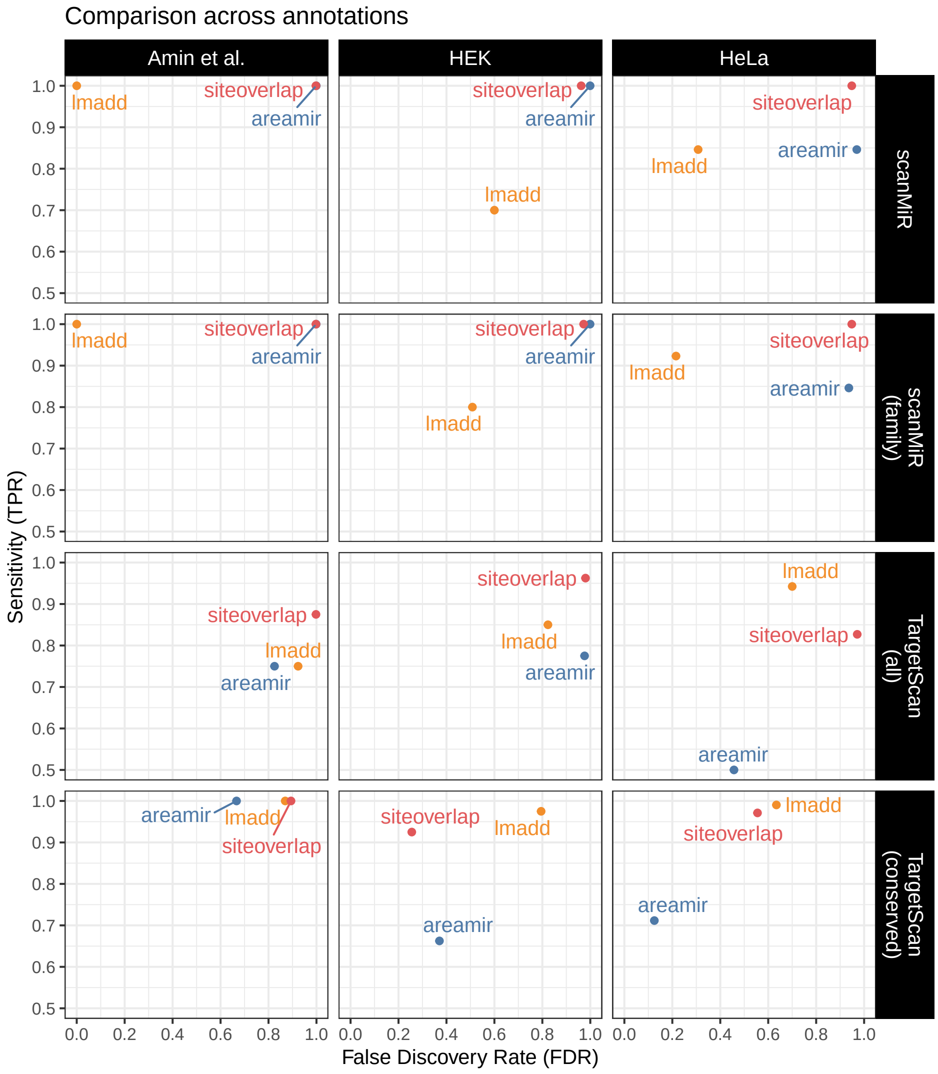

Supplementary Figure S6

Benchmark performance of the statistical tests using different target annotations. Applicability of the top tests to scanMiR-based annotations, and comparison of the two annotations. Of note, the best tests using the conserved TargetScan annotation perform poorly with larger target annotations; for scanMiR annotations we recommend the lmadd test.

Supplementary Figure S7

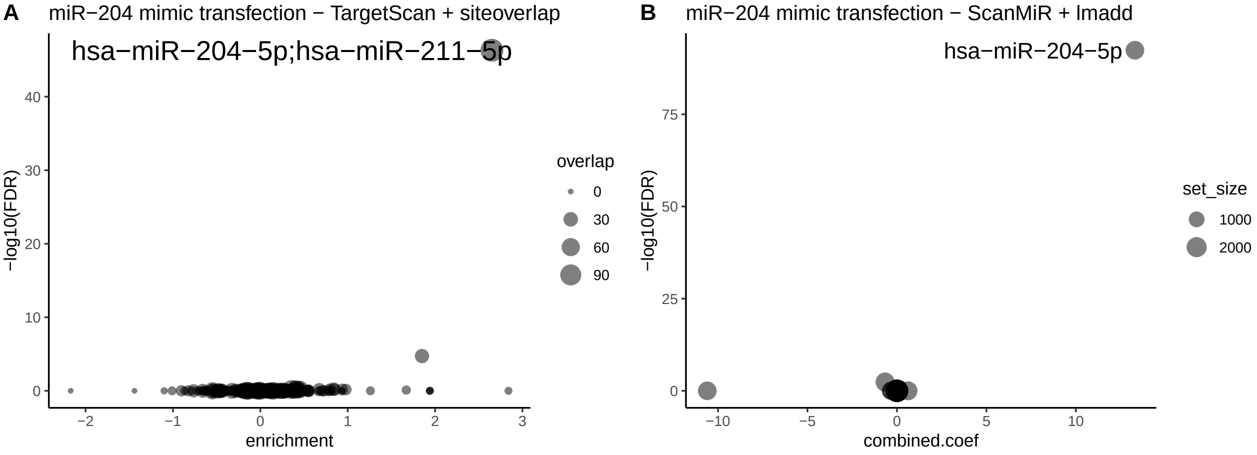

Supplementary Figure S7

Example *enrichMiR* analysis on a miR-204 overexpression dataset. A: Enrichment plot showing the results of an *enrichMiR* analysis on a DEA generated from RNA-sequencing data upon miR-204 overexpression in HEK cells using the siteoverlap test and the TargetScan human conserved annotation. Of note, with the TargetScan annotation it's not possible to disentangle individual microRNA family members since the common seed sequence (in this case 'UCCCUUU') is used to identify binding sites. **B:** Enrichment plot of the same *enrichMiR* analysis, however employing the lmadd test together with the scanMiR human binding site annotation. Since scanMiR identifies miRNA binding sites based on 12mer nucleotide sequences, using this annotation enables identifying individual microRNAs possibly involved in regulating the cellular response (as in this case the correct candidate miR-204-5p used for the overexpression).

Supplementary Figure S8

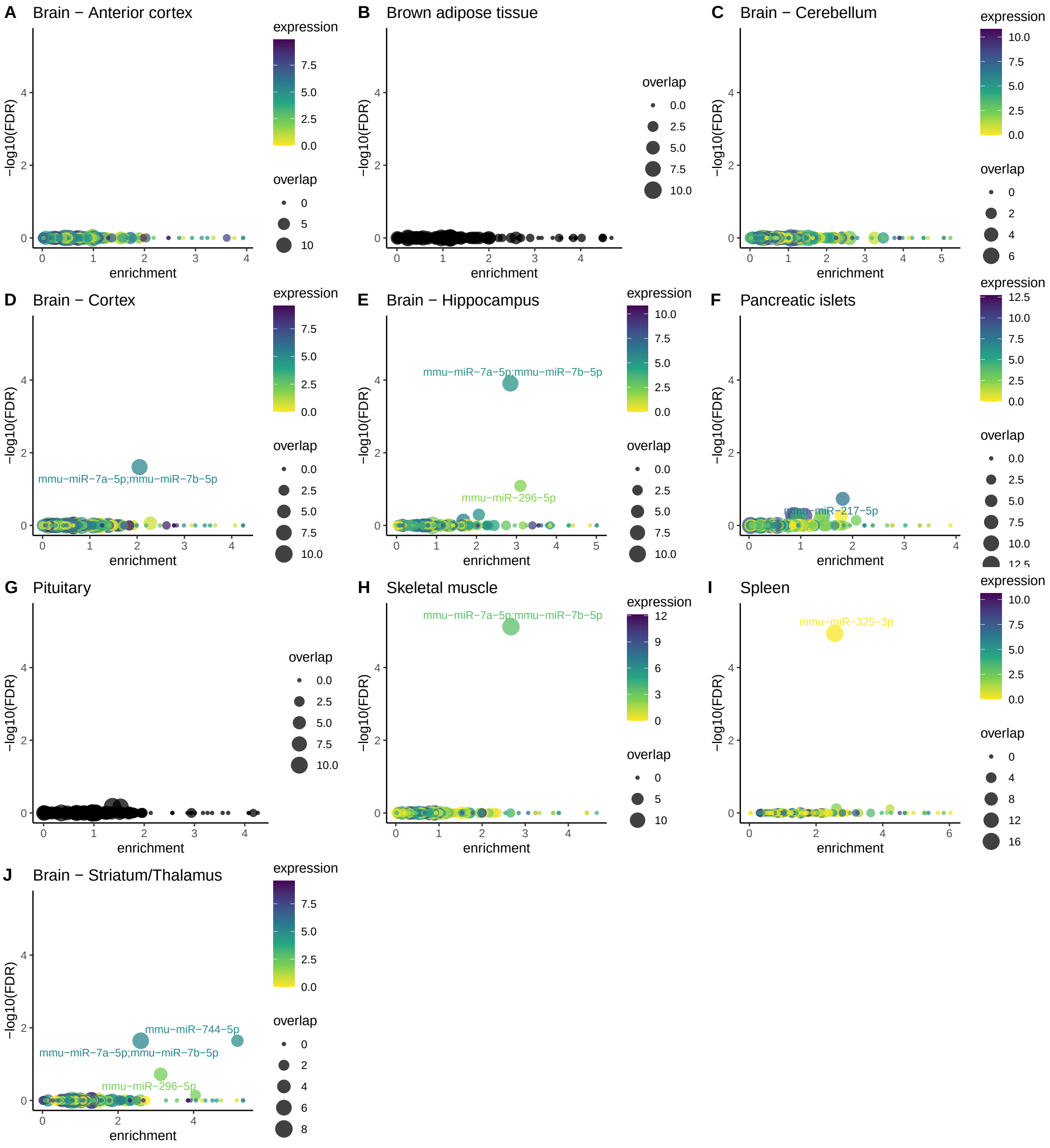

Supplementary Figure S8

***enrichMiR* analyses on RNA-sequencing datasets generated from several mouse tissues upon cyrano knockout. A-J:** Enrichment plots on the individual DEA with genes expressed higher than a baseMean of 10. Dots correspond to miRNA families including a colour coded expression of its top expressed member where applicable. miRNA expresion values were applied using the preset mouse miRNA expression tables provided in *enrichMiR* with the following specifications: **(A,D-E,J)** “Brain”, **(F)** “Pancreas”, **(H)** “Muscle” & **(I)** “Spleen”. In agreement with the original study performed by Kleaveland et al. (2018) (10), the *enrichMiR* analysis suggests in increase in miR-7 activity in “Cortex”, “Hippocampus”, “Skeletal Muscle” and “Striatum/Thalamus”. In contrast to the analysis performed by Kleaveland et al., the siteoverlap test performed with *enrichMiR* does not yield a significant increase in miR-7 acitivity in the “Anterior cortex”, “Pancreatic Islets” and “Pituitary”. Given that Kleaveland et al. used the Mann-Whitney test for their analysis and didn’t see a significant increase of miR-7 levels in “Pancreatic Islets” and “Pituitary”, we suspect that these differences come from the more robust error control of the siteoverlap test, as shown in the benchmarking.

Supplementary Figure S9

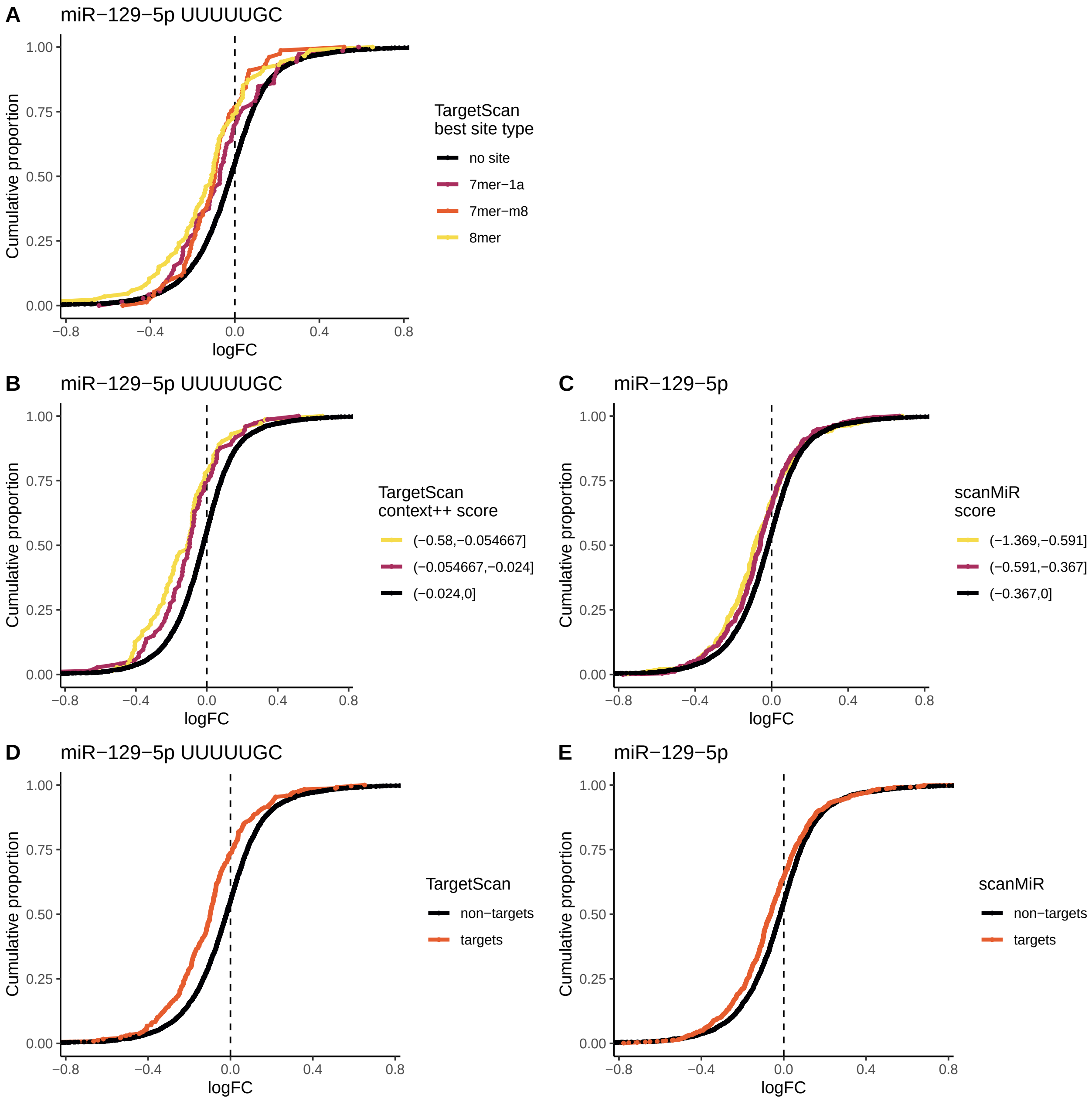

Supplementary Figure S9

CD plots supporting a potential role for miR-129-5p in PTX mediated homeostatic scaling in neurons. Analyses were performed on the 5'000 highest expressed genes of the RNA-sequencing dataset (as in Fig. 3). **A:** CD plot with the TargetScan rat conserved annotation, split by best site type (genes listed as "no sites" don't conatin a conserved binding site) **B:** CD plot split by score with the TargetScan rat conserved annotation. **C:** CD plot split by score with the scanMiR rat annotation. **D-E:** CD plots split in targets / non-targets with again the TargetScan rat conserved ("non-targets" equals no conserved binding site) and the scanMiR rat ("non-targets" equals no canonical 7mer or 8mer site) annotations.

Supplementary Figure S10

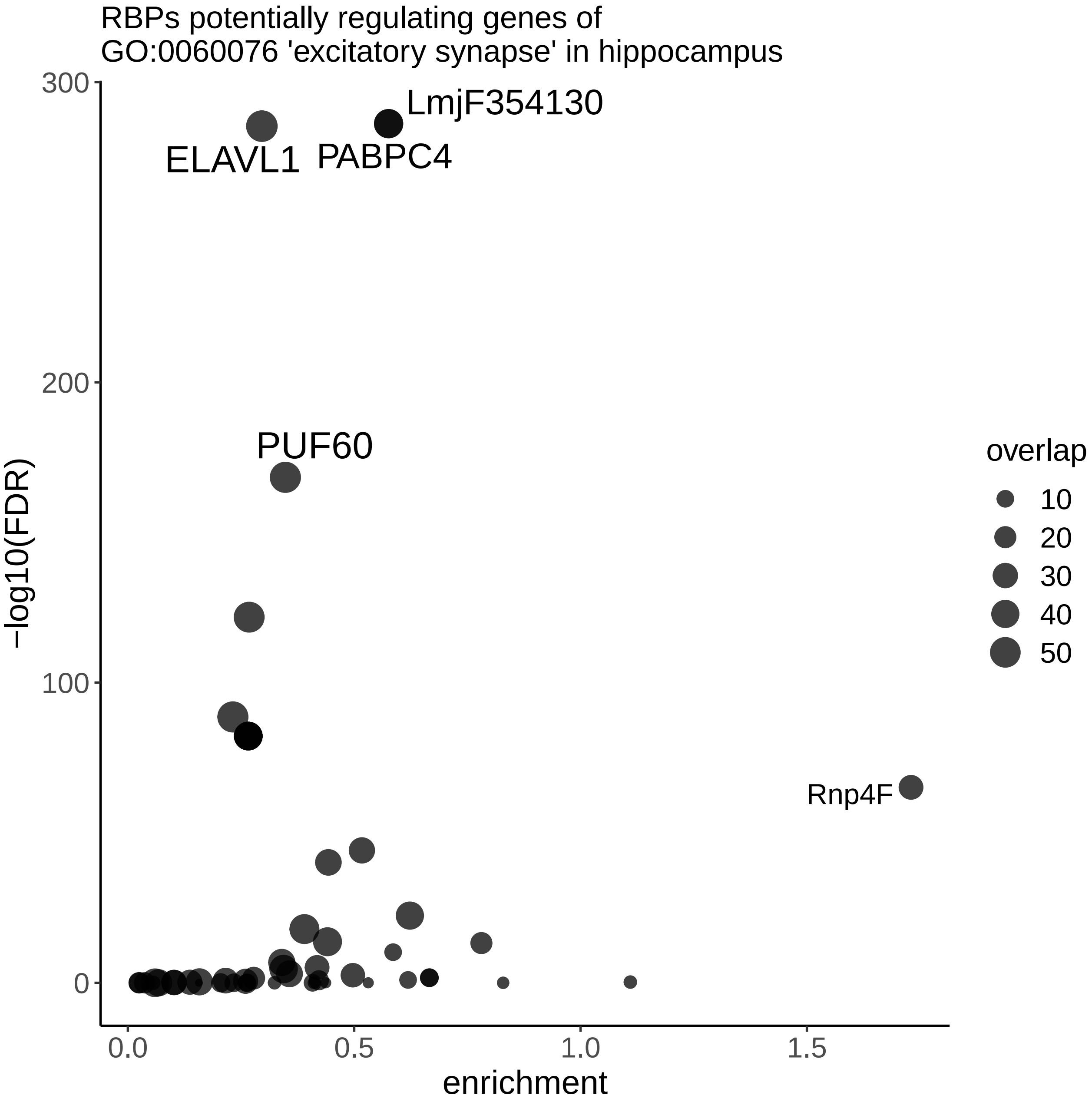

Supplementary Figure S10

***enrichMiR* analysis with the *oRNAment* RBP binding site collection.** Enrichment of RBP binding motifs in the 3'UTRs of mouse genes listed in the GO-Term "excitatory synapse" against genes expressed in the Hippocampus as reported in Lackinger et al. (2019) (17). Significance is assessed with the "siteoverlap" test. Notably, it has been previously suggested that neuronal-ELAV-like (nELAVL) RNA binding proteins associate with synaptic proteins in the human brain (18) and that nELAVL RBPs bind to the 3'UTRs of mRNAs (18, 19).
